# Supplementary material for: Inflammation and Organic Cation Transporters Novel (OCTNs)
Source: Biomolecules. 2024 Mar 25;14(4):392. doi: 10.3390/biom14040392 (PMC11048549; doi:10.3390/biom14040392)
Supplement: Supplementary file 1 [file biomolecules-14-00392-s001.zip › biomolecules-2896794-supplementary.pdf]

Table S1. ***SLC22A4*** **and** ***SLC22A5*** **SNPs and links with human pathologies**

Gene	SNP ID	SNP position	Gene/protein alteration	Pathology/Functional consequence	Reference
SLC22A4	rs1050152	Exon	L503F mutant	Increased transport of biguanides	[145]
SLC22A4	rs272893	Exon	I306T mutant	Reduced transport of gabapentin	[145]
SLC22A4	rs1050152	Exon	c.1507C>T (p.Leu503Phe)	Intestinal dysbiosis	[146]
SLC22A4	rs1050152	Exon	c.1507C>T (p.Leu503Phe)	CD	[147]
SLC22A4	rs1050152	Exon	c.1507C>T (p.Leu503Phe)	UC, Sporadic colorectal cancer; UC cases with cancer progression	[70]
SLC22A4	rs1050152	Exon	c.1507C>T (p.Leu503Phe)	CD	[148]
SLC22A4	rs1050152	Exon	c.1507C>T (p.Leu503Phe)	CD and IBD	[149]
SLC22A4/*SLC22A5*	rs1050152 / rs2631367	Exon / 5'-UTR	c.1507C>T (p.Leu503Phe) / c.-207C>T	Particularly refractory CD in the Slovenian population	[150]
SLC22A4/*SLC22A5*	rs1050152 / rs2631367 and rs2631372	Exon - 5'-UTR and Intron	c.1507C>T (p.Leu503Phe) - c.-207C>T and NC_000005.10: 132367885:G>C,T	Predictor of time to progression in gastrointestinal stromal tumours (GIST) patients receiving imatinib therapy	[151]
SLC22A4	rs1050152 and rs11950562	Exon and Intron	c.1507C>T (p.Leu503Phe) and NC_000005.10: 132316835:A>C,T	Associated with lower C5-OH levels are also associated with an increased risk for CD	[152]
SLC22A4	rs1050152 and rs3792876	Exon and Intron	c.1507C>T (p.Leu503Phe) and NC_000005.10:132301615:C>T	CD susceptibility in Australian paediatric cohort	[153]
SLC22A4/*SLC22A5*	rs1050152/ rs2631367	Exon /5′-UTR	c.1507C>T (p.Leu503Phe)/ -207G>C	Associated with susceptibility to CD in children	[154]
SLC22A4/*SLC22A5*	rs1050152/ rs2631367	Exon /5′-UTR	c.1507C>T (p.Leu503Phe)/ -207G>C	Association with the SNP IGR2096a_1, lying p-telomeric to SLC22A4 and SLC22A5 in Swedish population.	[155]
SLC22A4/*SLC22A5*	rs1050152/ rs2631367	Exon /	c.1507C>T (p.Leu503Phe) / c.-207C>T	Association with CD	[156]

SLC22A5	rs114269482	Exon	c.695C>T (p.Thr232Met)	PCD	[157]
SLC22A4	rs11568506	Intron	NC_000005.10: 132335766:G>A	Significantly associated with psoriasis risk	[158]
SLC22A5	rs11568520	Exon	c.51C>G (p.Phe17Leu)	PCD	[159]
SLC22A5	rs1157198543	Exon	c.1520T>C (p.Leu507Ser)	PCD	[160]
SLC22A5	rs11746555 and rs17622208	Intron and 5'-UTR	NC_000005.10: 132391340:G>A,C and NC_000005.10: 132381357:G>A,T	Associated with lower C5-OH levels are also associated with an increased risk for CD	[152]
SLC22A5	rs1178584184	Exon	c.428C>T (p.Pro143Leu)	PCD	[161]
SLC22A5	rs121908888	Exon	c.632A>G (p.Tyr211Cys)	PCD	[162]
SLC22A5	rs121908889	Exon	c.506G>A (p.Arg193Pro)	PCD	[163]
SLC22A5	rs121908889	Exon	c.506G>A (p.Arg193Gln)	PCD	[164]
SLC22A5	rs121908890	Exon	c.505C>T (p.Arg193Trp)	PCD	[165]
SLC22A5	rs121908891	Exon	c.1196G>A (p.Arg399Gln)	PCD	[166]
SLC22A5	rs1385634398	Exon	c.1064C>T (p.Ser379Leu)	PCD	[160]
SLC22A5	rs1408166345	Exon	c.1364C>G (p.Pro455Arg)	PCD	[160]
SLC22A5	rs144547521	Exon	c.1193C>T (p.Pro398Leu)	PCD	[160]
SLC22A5	rs145068530	Exon	c.601A>G (p.Met201Val)	PCD	[160]
SLC22A5	rs1457258524	Exon	c.700G>C (p.Gly258Arg)	PCD	[161]
SLC22A5	rs151231558	Exon	c.424G>A (p.Ala166Ser)	PCD	[167]
SLC22A5	rs185551386	Exon	c.680G>A (p.Arg227Leu)	PCD	[160]
SLC22A5	rs199689597	Exon	c.131C>G (p.Ala44Val)	PCD	[163]
SLC22A5	rs201082652	Exon	c.364G>A (p.Asp122Tyr)	PCD	[160]
SLC22A5	rs201262157	Exon	c.791C>T (p.Thr264Arg)	PCD	[163]
SLC22A5	rs202088921	Exon	c.136C>G (p.Pro46Ala)	PCD	[168]
SLC22A5	rs2073643	Intron	NC_000005.10: 132387595:T>C,G	In combination with other genes modulate spirometric indices	[169]
SLC22A4	rs2073838	Intron	NC_000005.10: 132313528:G>A,C	Associated with susceptibility to RA in	[170,171]

				East Asians but not in Europeans	
SLC22A4	rs2073838	Intron	NC_000005.10: 132313528:G>A,C	Associated with SUMO4 and CARD 15 in RA	[172]
SLC22A4	rs2306772	Intron	NC_000005.10: 132340287:C>T	The interaction with PTPN22 SNP as risk factor for RA	[173]
SLC22A5	rs2631367	5'-UTR	c.-207C>G	Extra-articular manifestations of Rheumatoid Arthritis (RA)	[174]
SLC22A5	rs2631367	5'-UTR	c.-207C>T	CD	[148]
SLC22A5	rs2631368	Promoter	-368T > G	Significantly associated with steroid resistance in Japanese patients with CD	[175]
SLC22A5	rs2631372	Intron	NC_000005.10: 132367885:G>C,T	Association with Inflammatory Bowel Disease (IBD) pathogenesis	[150]
SLC22A5	rs267607052	Exon	c.43G>T (p.Gly15Trp)	PCD	[167]
SLC22A5	rs267607053	Exon	c.1324_1325delinsAT (p.Ala442Ile)	PCD	[160]
SLC22A5	rs267607054	Exon	c.1195C>T (p.Arg399Trp)	PCD	[160]
SLC22A4	rs272893	Exon	c.917T>C,G (p.Ile306Thr)	Type 2 diabetes in Chinese Han patients	[176]
SLC22A4	rs272893	Exon	c.917T>C,G (p.Ile306Thr)	Association with rs273900 in CD	[177]
SLC22A4	rs273909	Intron	NC_000005.10: 132331659:A>G	In association with TOMM40 may be susceptibility locus for ischemic stroke in Japanese individuals	[178
SLC22A5	rs27437	Enhancer region	NC_000005.10: 132101267:A>G,T	Down-regulation in colorectal cancer (CRC)	[179]
SLC22A4	rs35260072	Intron	g.5708A>C	Crohn's disease (CD)	[180]
SLC22A5	rs377216516	Exon	c.1462C>G (p.Arg488Cys)	PCD	[160]
SLC22A5	rs377767445	Exon	c.137C>T (p.Pro46Leu)	PCD	[163]
SLC22A5	rs377767450	Exon	c.287G>C (p.Gly96Ala)	PCD	[160]
SLC22A4	rs3792876	Intron	NC_000005.10: 132301615:C>T	Increased risk of type I psoriasis	[181]
SLC22A4	rs3792876	Intron	NC_000005.10: 132301615:C>T	Statistically associated with pediatric-onset CD	[182]
SLC22A5	rs386134190	Exon	c.278C>T (p.Ser93Trp)	PCD	[163]

SLC22A5	rs386134191	Exon	c.283C>T (p.Leu95Val)	PCD	[163]
SLC22A5	rs386134192	Exon	c.344A>G (p.Asp115Gly)	PCD	[163]
SLC22A5	rs386134197	Exon	c.557T>C (p.Leu210Pro)	PCD	[160]
SLC22A5	rs386134198	Exon	c.629A>G (p.Asn210Ser)	PCD	[163]
SLC22A5	rs386134203	Exon	c.769C>T (p.Arg257Trp)	PCD	[163]
SLC22A5	rs386134205	Exon	c.746C>T (p.Ser225Leu)	PCD	[163]
SLC22A5	rs386134206	Exon	c.692C>T (p.Ser231Cys)	PCD	[163]
SLC22A5	rs386134208	Exon	c.839C>A (p.Ser280Tyr)	PCD	[183]
SLC22A5	rs386134210	Exon	c.845G>C (p.Arg282Gln)	PCD	[183]
SLC22A5	rs386134211	Exon	c.849G>T (p.Trp283Cys)	PCD	[184]
SLC22A5	rs386134214	Exon	c.1088T>C (p.Leu363His)	PCD	[163]
SLC22A5	rs386134218	Exon	c.1340A>G (p.Tyr447Cys)	PCD	[183]
SLC22A5	rs386134219	Exon	c.1342G>A (p.Val448Met)	PCD	[163]
SLC22A5	rs386134222	Exon	c.1409C>T (p.Ser470Phe)	PCD	[163]
SLC22A5	rs386134223	Exon	c.1412G>A (p.Arg471Leu)	PCD	[163]
SLC22A4-SLC22A5	rs460089-GC_rs2631365-TC	Promoter	NC_000005.10: 132294078:C>A,G	Increased response to imatinib treatment	[185]
SLC22A5	rs60376624	Exon	c.1400C>G (p.Ser467Cys)	PCD	[157,160]
SLC22A5	rs61731073	Exon	c.1072T>A (p.Tyr358Asn)	PCD	[160]
SLC22A5	rs68018207	Exon	c.1051T>C (p.Trp351Arg)	PCD	[183]
SLC22A5	rs72552723	Exon	c.56G>A (p.Arg19Pro)	PCD	[166]
SLC22A5	rs72552724	Exon	c.83G>T (p.Ser28Ile)	PCD	[163]
SLC22A5	rs72552725	Exon	c.95A>G (Asn32Ser)	PCD	[168]
SLC22A5	rs72552726	Exon	c.248G>T (p.Arg83Leu)	PCD	[186]
SLC22A5	rs72552728	Exon	c.725G>T (p.Gly242Val)	PCD	[165]
SLC22A5	rs72552729	Exon	c.847T>C (p.Trp283Arg)	PCD	[187]
SLC22A5	rs72552730	Exon	c.902C>A (p.Ala301Asp)	PCD	[165]
SLC22A5	rs72552732	Exon	c.1319C>T (p.Thr440Met)	PCD	[160]

SLC22A5	rs72552733	Exon	c.1336G>T (p.Val446Phe)	PCD	[183]
SLC22A5	rs72552734	Exon	c.1354G>A (p.Glu452Lys)	PCD	[160]
SLC22A5	rs72552735	Exon	c.1433C>T (p.Pro478Leu)	PCD	[163]
SLC22A5	rs748605096	Exon	c.368T>G (p.Val123Gly)	PCD	[160]
SLC22A5	rs749282641	Exon	c.1411C>A (p.Arg471Cys)	PCD	[161]
SLC22A5	rs756650860	Exon	c.688T>C (p.Phe230Leu)	PCD	[160]
SLC22A5	rs757711838	Exon	c.224G>A (p.Arg75Pro)	PCD	[160][17]
SLC22A4	rs768484124	Exon	c.338G>A (p.Cys113Tyr)	Hearing loss	[188]
SLC22A5	rs772578415	Exon	c.77G>A (p.Ser26Asn)	PCD	[183]
SLC22A5	rs781721860	Exon	c.595G>A (p.Val175Met)	PCD	[163]
SLC22A5	rs796052033	Exon	c.614T>A (p.Met205Arg)	PCD	[163]
SLC22A5	rs886042092	Exon	c.1085C>T (p.Ser362Leu)	PCD	[161]
SLC22A5	UniProt: VAR_064114	Exon	p.Thr66Pro	PCD	[160]
SLC22A5	UniProt: VAR_064130	Exon	p.Ala240Thr	PCD	[160]
SLC22A5	UniProt: VAR_064146	Exon	p.Phe443Val	PCD	[160]
SLC22A5	UniProt: VAR_066845	Exon	p.Arg471Pro	PCD	[183]
SLC22A5	UniProt: VAR_079641	Exon	p.Pro16Leu	PCD	[163]
SLC22A5	UniProt: VAR_079646	Exon	p.Cys50Tyr	PCD	[163]
SLC22A5	UniProt: VAR_079662	Exon	p.Pro247Arg	PCD	[163]
SLC22A5	UniProt: VAR_079679	Exon	p.Val439Gly	PCD	[163]
SLC22A5	UniProt: VAR_079681	Exon	p.Gly462Val		[163]
SLC22A5	UniProt: VAR_079684	Exon	p.Leu476Arg	PCD	[163]

UC: Ulcerative Colitis; CD: Crohn's disease; RA: Rheumatoid Arthritis; IBD: Inflammatory Bowel Disease; PCD: primary carnitine deficiency

70. Martini, M.; Ferrara, A.M.; Giachelia, M.; Panieri, E.; Siminovitch, K.; Galeotti, T.; Larocca, L.M.; Pani, G. Association of the OCTN1/1672T variant with increased risk for colorectal cancer in young individuals and ulcerative colitis patients. *Inflamm Bowel Dis* **2012**, *18*, 439-448, doi:10.1002/ibd.21814.

145. Futatsugi, A.; Masuo, Y.; Kawabata, S.; Nakamichi, N.; Kato, Y. L503F variant of carnitine/organic cation transporter 1 efficiently transports metformin and other biguanides. *J Pharm Pharmacol* **2016**, *68*, 1160-1169, doi:10.1111/jphp.12574.

146. Petito, V.; Fidaleo, M.; Pani, G.; Putignani, L.; Gasbarrini, A.; Scaldaferri, F. Tumor necrosis factor-alpha and solute carrier family 22 member 4 gene polymorphisms as potential determinants of intestinal dysbiosis. *Dig Liver Dis* **2020**, *52*, 691-693, doi:10.1016/j.dld.2020.03.012.
147. Lee, Y.H.; Song, G.G. Pathway analysis of a genome-wide association study of ileal Crohn's disease. *DNA Cell Biol* **2012**, *31*, 1549-1554, doi:10.1089/dna.2012.1605.
148. Xuan, C.; Zhang, B.B.; Yang, T.; Deng, K.F.; Li, M.; Tian, R.J. Association between OCTN1/2 gene polymorphisms (1672C-T, 207G-C) and susceptibility of Crohn's disease: a meta-analysis. *Int J Colorectal Dis* **2012**, *27*, 11-19, doi:10.1007/s00384-011-1265-x.
149. Lin, Z.; Nelson, L.; Franke, A.; Poritz, L.; Li, T.Y.; Wu, R.; Wang, Y.; MacNeill, C.; Thomas, N.J.; Schreiber, S., et al. OCTN1 variant L503F is associated with familial and sporadic inflammatory bowel disease. *J Crohns Colitis* **2010**, *4*, 132-138, doi:10.1016/j.crohns.2009.09.003.
150. Repnik, K.; Potocnik, U. Haplotype in the IBD5 region is associated with refractory Crohn's disease in Slovenian patients and modulates expression of the SLC22A5 gene. *J Gastroenterol* **2011**, *46*, 1081-1091, doi:10.1007/s00535-011-0426-6.
151. Angelini, S.; Pantaleo, M.A.; Ravegnini, G.; Zenesini, C.; Cavrini, G.; Nannini, M.; Fumagalli, E.; Palassini, E.; Saponara, M.; Di Battista, M., et al. Polymorphisms in OCTN1 and OCTN2 transporters genes are associated with prolonged time to progression in unresectable gastrointestinal stromal tumours treated with imatinib therapy. *Pharmacol Res* **2013**, *68*, 1-6, doi:10.1016/j.phrs.2012.10.015.
152. Ryckman, K.K.; Smith, C.J.; Jelliffe-Pawlowski, L.L.; Momany, A.M.; Berberich, S.L.; Murray, J.C. Metabolic heritability at birth: implications for chronic disease research. *Hum Genet* **2014**, *133*, 1049-1057, doi:10.1007/s00439-014-1450-4.
153. Wagner, J.; Sim, W.H.; Ellis, J.A.; Ong, E.K.; Catto-Smith, A.G.; Cameron, D.J.; Bishop, R.F.; Kirkwood, C.D. Interaction of Crohn's disease susceptibility genes in an Australian paediatric cohort. *PLoS One* **2010**, *5*, e15376, doi:10.1371/journal.pone.0015376.
154. Cucchiara, S.; Latiano, A.; Palmieri, O.; Staiano, A.M.; D'Inca, R.; Guariso, G.; Vieni, G.; Rutigliano, V.; Borrelli, O.; Valvano, M.R., et al. Role of CARD15, DLG5 and OCTN genes polymorphisms in children with inflammatory bowel diseases. *World J Gastroenterol* **2007**, *13*, 1221-1229, doi:10.3748/wjg.v13.i8.1221.
155. Torkvist, L.; Noble, C.L.; Lordal, M.; Sjoqvist, U.; Lindforss, U.; Nimmo, E.R.; Lofberg, R.; Russell, R.K.; Satsangi, J. Contribution of the IBD5 locus to Crohn's disease in the Swedish population. *Scand J Gastroenterol* **2007**, *42*, 200-206, doi:10.1080/00365520600842278.
156. Silverberg, M.S.; Duerr, R.H.; Brant, S.R.; Bromfield, G.; Datta, L.W.; Jani, N.; Kane, S.V.; Rotter, J.I.; Philip Schumm, L.; Hillary Steinhart, A., et al. Refined genomic localization and ethnic differences observed for the IBD5 association with Crohn's disease. *Eur J Hum Genet* **2007**, *15*, 328-335, doi:10.1038/sj.ejhg.5201756.
157. Dobrowolski, S.F.; McKinney, J.T.; Amat di San Filippo, C.; Giak Sim, K.; Wilcken, B.; Longo, N. Validation of dye-binding/high-resolution thermal denaturation for the identification of mutations in the SLC22A5 gene. *Hum Mutat* **2005**, *25*, 306-313, doi:10.1002/humu.20137.
158. Li, Y.; Chang, M.; Schrodi, S.J.; Callis-Duffin, K.P.; Matsunami, N.; Civello, D.; Bui, N.; Catanese, J.J.; Leppert, M.F.; Krueger, G.G., et al. The 5q31 variants associated with psoriasis and Crohn's disease are distinct. *Hum Mol Genet* **2008**, *17*, 2978-2985, doi:10.1093/hmg/ddn196.
159. Urban, T.J.; Gallagher, R.C.; Brown, C.; Castro, R.A.; Lagpacan, L.L.; Brett, C.M.; Taylor, T.R.; Carlson, E.J.; Ferrin, T.E.; Burchard, E.G., et al. Functional genetic diversity in the high-affinity carnitine transporter OCTN2 (SLC22A5). *Mol Pharmacol* **2006**, *70*, 1602-1611, doi:10.1124/mol.106.028126.
160. Li, F.Y.; El-Hattab, A.W.; Bawle, E.V.; Boles, R.G.; Schmitt, E.S.; Scaglia, F.; Wong, L.J. Molecular spectrum of SLC22A5 (OCTN2) gene mutations detected in 143 subjects evaluated for systemic carnitine deficiency. *Hum Mutat* **2010**, *31*, E1632-1651, doi:10.1002/humu.21311.
161. Lee, N.C.; Tang, N.L.; Chien, Y.H.; Chen, C.A.; Lin, S.J.; Chiu, P.C.; Huang, A.C.; Hwu, W.L. Diagnoses of newborns and mothers with carnitine uptake defects through newborn screening. *Mol Genet Metab* **2010**, *100*, 46-50, doi:10.1016/j.ymgme.2009.12.015.
162. Vaz, F.M.; Scholte, H.R.; Ruiter, J.; Hussaarts-Odijk, L.M.; Pereira, R.R.; Schweitzer, S.; de Klerk, J.B.; Waterham, H.R.; Wanders, R.J. Identification of two novel mutations in OCTN2 of three patients with systemic carnitine deficiency. *Hum Genet* **1999**, *105*, 157-161, doi:10.1007/s004399900105.
163. Frigeni, M.; Balakrishnan, B.; Yin, X.; Calderon, F.R.O.; Mao, R.; Pasquali, M.; Longo, N. Functional and molecular studies in primary carnitine deficiency. *Hum Mutat* **2017**, *38*, 1684-1699, doi:10.1002/humu.23315.
164. Burwinkel, B.; Kreuder, J.; Schweitzer, S.; Vorgerd, M.; Gempel, K.; Gerbitz, K.D.; Kilimann, M.W. Carnitine transporter OCTN2 mutations in systemic primary carnitine deficiency: a novel Arg169Gln mutation and a recurrent Arg282ter

mutation associated with an unconventional splicing abnormality. *Biochem Biophys Res Commun* **1999**, *261*, 484-487, doi:10.1006/bbrc.1999.1060.
165. Wang, Y.; Taroni, F.; Garavaglia, B.; Longo, N. Functional analysis of mutations in the OCTN2 transporter causing primary carnitine deficiency: lack of genotype-phenotype correlation. *Hum Mutat* **2000**, *16*, 401-407, doi:10.1002/1098-1004(200011)16:5<401::AID-HUMU4>3.0.CO;2-J.
166. Wang, Y.; Korman, S.H.; Ye, J.; Gargus, J.J.; Gutman, A.; Taroni, F.; Garavaglia, B.; Longo, N. Phenotype and genotype variation in primary carnitine deficiency. *Genet Med* **2001**, *3*, 387-392, doi:10.1097/00125817-200111000-00002.
167. El-Hattab, A.W.; Li, F.Y.; Shen, J.; Powell, B.R.; Bawle, E.V.; Adams, D.J.; Wahl, E.; Kobori, J.A.; Graham, B.; Scaglia, F., et al. Maternal systemic primary carnitine deficiency uncovered by newborn screening: clinical, biochemical, and molecular aspects. *Genet Med* **2010**, *12*, 19-24, doi:10.1097/GIM.0b013e3181c5e6f7.
168. Schimmenti, L.A.; Crombez, E.A.; Schwahn, B.C.; Heese, B.A.; Wood, T.C.; Schroer, R.J.; Bentler, K.; Cederbaum, S.; Sarafoglou, K.; McCann, M., et al. Expanded newborn screening identifies maternal primary carnitine deficiency. *Mol Genet Metab* **2007**, *90*, 441-445, doi:10.1016/j.ymgme.2006.10.003.
169. Tang, M.F.; Sy, H.Y.; Kong, A.P.; Ko, F.W.; Wang, S.S.; Liu, T.C.; Chan, W.C.; Wong, G.W.; Hon, K.L.; Chan, J.C., et al. Genetic effects of multiple asthma loci identified by genomewide association studies on asthma and spirometric indices. *Pediatr Allergy Immunol* **2016**, *27*, 185-194, doi:10.1111/pai.12505.
170. Lee, Y.H.; Bae, S.C.; Kim, J.H.; Seo, Y.H.; Choi, S.J.; Ji, J.D.; Song, G.G. Meta-analysis of SLC22A4 and RUNX1 polymorphisms: Associations with rheumatoid arthritis susceptibility. *Z Rheumatol* **2015**, *74*, 351-358, doi:10.1007/s00393-014-1447-3.
171. Ren, T.L.; Han, Z.J.; Yang, C.J.; Hang, Y.X.; Fang, D.Y.; Wang, K.; Zhu, X.; Ji, X.J.; Zhou, F.F. Association of SLC22A4 gene polymorphism with Rheumatoid arthritis in the Chinese population. *J Biochem Mol Toxicol* **2014**, *28*, 206-210, doi:10.1002/jbt.21554.
172. Ding, Y.; Cong, L.; Ionita-Laza, I.; Lo, S.H.; Zheng, T. Constructing gene association networks for rheumatoid arthritis using the backward genotype-trait association (BGTA) algorithm. *BMC Proc* **2007**, *1 Suppl 1*, S13, doi:10.1186/1753-6561-1-s1-s13.
173. Jung, J.; Song, J.J.; Kwon, D. Allelic based gene-gene interactions in rheumatoid arthritis. *BMC Proc* **2009**, *3 Suppl 7*, S76, doi:10.1186/1753-6561-3-S7-S76.
174. Pawlik, A.; Paradowska-Gorycka, A.; Safranow, K.; Dziedziejko, V.; Dutkiewicz, G.; Slucznowska-Glabowska, S.; Juzyszyn, Z.; Drozdzik, M. SLC22A5 polymorphism associated with risk of extra-articular manifestations in rheumatoid arthritis patients. *Reumatologia* **2019**, *57*, 3-7, doi:10.5114/reum.2019.83233.
175. Nakahara, S.; Arimura, Y.; Saito, K.; Goto, A.; Motoya, S.; Shinomura, Y.; Miyamoto, A.; Imai, K. Association of SLC22A4/5 polymorphisms with steroid responsiveness of inflammatory bowel disease in Japan. *Dis Colon Rectum* **2008**, *51*, 598-603, doi:10.1007/s10350-008-9208-5.
176. Long, G.; Zhang, G.; Zhang, F.; Ye, D.; Yang, D.; Yang, Y. Relationship Between SLC22A1 and SLC22A4 Gene Polymorphisms and Risk of Type 2 Diabetes in Chinese Han Population. *Clin Lab* **2018**, *64*, 1357-1361, doi:10.7754/Clin.Lab.2018.180129.
177. Weersma, R.K.; Zhou, L.; Nolte, I.M.; van der Steege, G.; van Dullemen, H.M.; Oosterom, E.; Bok, L.; Peppelenbosch, M.P.; Faber, K.N.; Kleibeuker, J.H., et al. Runt-related transcription factor 3 is associated with ulcerative colitis and shows epistasis with solute carrier family 22, members 4 and 5. *Inflamm Bowel Dis* **2008**, *14*, 1615-1622, doi:10.1002/ibd.20610.
178. Yamase, Y.; Horibe, H.; Ueyama, C.; Fujimaki, T.; Oguri, M.; Kato, K.; Arai, M.; Watanabe, S.; Yamada, Y. Association of TOMM40 and SLC22A4 polymorphisms with ischemic stroke. *Biomed Rep* **2015**, *3*, 491-498, doi:10.3892/br.2015.457.
179. Zou, D.; Lou, J.; Ke, J.; Mei, S.; Li, J.; Gong, Y.; Yang, Y.; Zhu, Y.; Tian, J.; Chang, J., et al. Integrative expression quantitative trait locus-based analysis of colorectal cancer identified a functional polymorphism regulating SLC22A5 expression. *Eur J Cancer* **2018**, *93*, 1-9, doi:10.1016/j.ejca.2018.01.065.
180. Sebastian-delaCruz, M.; Olazagoitia-Garmendia, A.; Gonzalez-Moro, I.; Santin, I.; Garcia-Etxebarria, K.; Castellanos-Rubio, A. Implication of m6A mRNA Methylation in Susceptibility to Inflammatory Bowel Disease. *Epigenomes* **2020**, *4*, doi:10.3390/epigenomes4030016.
181. Prieto-Perez, R.; Solano-Lopez, G.; Cabaleiro, T.; Roman, M.; Ochoa, D.; Talegon, M.; Baniandres, O.; Lopez-Estebaranz, J.L.; de la Cueva, P.; Dauden, E., et al. Polymorphisms Associated with Age at Onset in Patients with Moderate-to-Severe Plaque Psoriasis. *J Immunol Res* **2015**, *2015*, 101879, doi:10.1155/2015/101879.
182. de Ridder, L.; Weersma, R.K.; Dijkstra, G.; van der Steege, G.; Benninga, M.A.; Nolte, I.M.; Taminiau, J.A.; Hommes, D.W.; Stokkers, P.C. Genetic susceptibility has a more important role in pediatric-onset Crohn's disease than in adult-onset Crohn's disease. *Inflamm Bowel Dis* **2007**, *13*, 1083-1092, doi:10.1002/ibd.20171.

183. Rose, E.C.; di San Filippo, C.A.; Ndukwe Erlingsson, U.C.; Ardon, O.; Pasquali, M.; Longo, N. Genotype-phenotype correlation in primary carnitine deficiency. *Hum Mutat* **2012**, *33*, 118-123, doi:10.1002/humu.21607.
184. Koizumi, A.; Nozaki, J.; Ohura, T.; Kayo, T.; Wada, Y.; Nezu, J.; Ohashi, R.; Tamai, I.; Shoji, Y.; Takada, G., et al. Genetic epidemiology of the carnitine transporter OCTN2 gene in a Japanese population and phenotypic characterization in Japanese pedigrees with primary systemic carnitine deficiency. *Hum Mol Genet* **1999**, *8*, 2247-2254, doi:10.1093/hmg/8.12.2247.
185. Jaruskova, M.; Curik, N.; Hercog, R.; Polivkova, V.; Motlova, E.; Benes, V.; Klamova, H.; Pecherkova, P.; Belohlavkova, P.; Vrbacky, F., et al. Genotypes of SLC22A4 and SLC22A5 regulatory loci are predictive of the response of chronic myeloid leukemia patients to imatinib treatment. *J Exp Clin Cancer Res* **2017**, *36*, 55, doi:10.1186/s13046-017-0523-3.
186. Makhseed, N.; Vallance, H.D.; Potter, M.; Waters, P.J.; Wong, L.T.; Lillquist, Y.; Pasquali, M.; Amat di San Filippo, C.; Longo, N. Carnitine transporter defect due to a novel mutation in the SLC22A5 gene presenting with peripheral neuropathy. *J Inherit Metab Dis* **2004**, *27*, 778-780, doi:10.1023/b:boli.0000045837.23328.f4.
187. Mayatepek, E.; Nezu, J.; Tamai, I.; Oku, A.; Katsura, M.; Shimane, M.; Tsuji, A. Two novel missense mutations of the OCTN2 gene (W283R and V446F) in a patient with primary systemic carnitine deficiency. *Hum Mutat* **2000**, *15*, 118, doi:10.1002/(SICI)1098-1004(200001)15:1<118::AID-HUMU28>3.0.CO;2-8.
188. Ben Said, M.; Grati, M.; Ishimoto, T.; Zou, B.; Chakchouk, I.; Ma, Q.; Yao, Q.; Hammami, B.; Yan, D.; Mittal, R., et al. A mutation in SLC22A4 encoding an organic cation transporter expressed in the cochlea strial endothelium causes human recessive non-syndromic hearing loss DFNB60. *Hum Genet* **2016**, *135*, 513-524, doi:10.1007/s00439-016-1657-7.
